# Interventions to promote resilience and passion for work in health settings: A mixed-methods systematic review

**DOI:** 10.1016/j.ijnsa.2024.100242

**Published:** 2024-09-21

**Authors:** Supan Unjai, Elizabeth M. Forster, Amy E. Mitchell, Debra K. Creedy

**Affiliations:** a School of Nursing and Midwifery, Nathan Campus, Griffith University, N48 Health Sciences Building, Level 2.06, 170 Kessels Road, QLD 4111, Australia; b Faculty of Nursing, Khon Kaen University, Thailand; c School of Nursing, Midwifery and Social Work, The University of Queensland, Australia; d Parenting and Family Support Centre, The University of Queensland, Australia; e Centre for Mental Health, Griffith University, Australia

**Keywords:** Resilience, Passion, Intervention, Healthcare workers, Systematic review

## Abstract

**Background:**

Resilience and passion for work are associated with better psychosocial wellbeing and professional quality of life for healthcare workers.

**Objective:**

To evaluate the characteristics and efficacy of interventions to promote resilience and passion for work in health settings.

**Methods:**

A comprehensive search was conducted across six databases (MEDLINE, EMBASE, CINAHL, Web of Science, Scopus, and PsycINFO) for articles published between January 2003 and February 2023. Studies utilizing both quantitative and qualitative methodologies were included. Methodological quality assessment was performed using the Mixed Methods Appraisal Tool. Data from the included studies were analyzed using a convergent mixed methods design.

**Results:**

A total of 33 studies met the inclusion criteria. All reported on interventions designed to enhance resilience for healthcare workers. None reported on interventions to enhance passion for work. Interventions included mindfulness-based programs, psychoeducation workshops, stress management techniques, and professional coaching. Interventions varied widely in terms of delivery modality, format, content, intensity, duration, and outcomes. Of the studies reporting quantitative data, most (21/29) reported statistically significant improvements in resilience. Of the studies reporting qualitative data, all reported a positive impact of the intervention on resilience and psychological well-being.

**Conclusions:**

Overall, interventions designed to enhance resilience in health care settings appear to be effective across a variety of healthcare settings. The diversity of effective intervention approaches, delivery formats, intensity and duration suggest that brief, light-touch or self-directed online interventions may be equally as effective as more intensive, lengthy, in-person or group-based interventions. This provides health care organisations with the opportunity to select and flexibly implement interventions that align with organisational, and staff needs and preferences. Future research needs to explore effective approaches to building passion for work.

## Tweetable abstract

A mixed-methods systematic review identified resilience-boosting interventions in health settings, such as mindfulness programs, psychoeducation workshops, stress management, and professional coaching. Notably, no interventions were reported for enhancing passion for work.

## What is already known

• Resilience and passion for work are crucial for enhancing quality of life among healthcare professionals, particularly in high-stress environments like intensive care units (ICUs).
• A growing body of research has investigated the effectiveness of resilience interventions for healthcare workers; however, there has been a lack of systematic review of these studies thus far.
• There has been no study specifically investigating passion for work among ICU healthcare professionals, though research in other healthcare settings has not been explored.

## What this paper adds

• Resilience-boosting interventions comprised mindfulness-based programs, psychoeducation workshops, stress management techniques, and professional coaching.
• Resilience interventions exhibited considerable variation in delivery mode, format, content, intensity, duration, and resulting outcomes.
• Documented interventions aimed at boosting passion for work in health settings were not found.

## 1 Introduction

Healthcare workers face numerous stressors and challenges in their workplace. Work-related stress can impact psychological well-being of staff working in health settings, contributing to compassion fatigue, depression, anxiety, decreased job satisfaction, burnout, and ultimately impacting the quality of patient care (Cavanagh et al., 2020; Sarafis et al., 2016; Yılmaz, 2017). In response to these challenges, building resilience and passion for work among healthcare workers has emerged as a promising strategy to promote well-being (Hasan and Alsulami, 2024; Unjai et al., 2023).

Resilience, defined as the ability to bounce back from adversity, adapt to change, and thrive in the face of challenges, is a critical asset for healthcare workers (Connor and Davidson, 2003). Promoting resilience in healthcare workers can help to protect against psychological distress in crisis situations and has implications for the quality of patient care and organizational effectiveness within healthcare settings (Ata et al., 2020; Baskin and Bartlett, 2021). Empirical research has identified links between healthcare worker resilience and a number of personal factors and psychological factors such as number of working hours per week, age, years of clinical experience, nationality, types of shifts worked, perceived health status, and perceived stress (Aldarmasi, 2022; Simões de Almeida et al., 2023; Unjai et al., 2022, 2023). However, no comprehensive study report has yet emerged to analyze the effectiveness of interventions aimed at promoting resilience among healthcare workers.

Passion is defined as a strong inclination towards an activity that an individual enjoys, believes is essential, and spends significant time and energy in doing (Špehar et al., 2016). Based on the Dualistic Model of Passion, passion for work can be divided into two categories: “obsessive passion”, which is related to a controlled internalization of the work activity in one's identity, and “harmonious passion”, which is concerned with the autonomous internalization of work activity (Vallerand and Houlfort, 2003). Harmonious and obsessive passion are seen as valuable resources, each leading to different outcomes. The study by Landay and colleagues (2022) expands upon the principles outlined in the conservation of resources theory, illustrating that harmonious passion indirectly reduces disengagement and exhaustion by decreasing job stress among nurses. However, obsessive passion did not yield significant effects (Landay et al., 2022. Similarly, Gkorezis et al. (2021) found that harmonious work passion among healthcare workers was positively related to intrinsic motivation.

In light of the growing body of literature supporting links between resilience and passion for work in the healthcare workforce, and enhanced psychological well-being especially professional quality of life (Unjai et al., 2023), a systematic review of the literature examining interventions to boost resilience and passion for work among healthcare workers is well overdue. While a previous systematic review assessed existing research on the effectiveness of resilience interventions among healthcare professionals, inadequate description of intervention characteristics may limit its utility in informing further intervention development (Cleary et al., 2018). Additionally, while evidence reports a positive relationship between harmonious passion and compassion satisfaction (a positive aspect of professional quality of life), international research on work passion interventions remains unexplored (Unjai et al., 2023).

To address these gaps in the literature, this systematic review adopts a mixed method approach to provide a comprehensive understanding of interventions to enhance resilience or passion for work among healthcare workers. By synthesizing both quantitative and qualitative evidence, this review aims to elucidate the efficacy, mechanisms, and limitations of these types of interventions in health settings. Specifically, the review seeks to answer the following research questions:

1. What are the characteristics of interventions used to promote resilience or passion for work in health settings?
2. What is the overall efficacy of interventions designed to promote resilience or passion for work in health settings?

## 2 Methods

### 2.1 Protocol

The protocol was prospectively registered in the International Prospective Register of Systematic Reviews (PROSPERO 2023 CRD42023394575).

### 2.2 Eligibility criteria

Inclusion and exclusion criteria are summarised in Table 1. This review considered all primary research using any study design, published between January 2003 and June 2023. Date limiters were applied to ensure searches captured recent evidence, and because the concept of “resilience” was first described in the academic literature in 2003. Studies with mixed participant samples (e.g., included medical/nursing students or health workers in non-registered occupations) were excluded unless data for the registered health professionals were reported separately. Studies that did not include a sufficiently clear report of intervention characteristics or lacked data indicating the efficacy of the intervention were excluded.

**Table 1:** Inclusion and exclusion criteria.

	Inclusion criteria	Exclusion criteria
General criteria	Published 2003–2023; English language; peer-reviewed journal	Published before 2003; not English language; not peer-reviewed
Population	All workers in health professional occupations	Medical students, nursing students, health workers in non-registered occupations such as health workers, peer support workers
Interventions	Interventions used to promote resilience and/or passion for work in health care settings	Study does not explicitly relate to resilience or passion for work; intervention designed for or delivered to workers with a diagnosed mental health condition
Outcomes	Reported characteristics or details of interventions; efficacy/effectiveness (e.g., change in resilience or passion for work scores, participant experiences of intervention)	No or unclear report of intervention characteristics or efficacy/effectiveness of intervention
Type of studies	Primary research of any study type and design (qualitative, quantitative, mixed methods)	Grey literature, summaries, commentaries, review documents, case studies, reviews

### 2.3 Information sources and search strategy

A systematic search of electronic databases including MEDLINE (via Ovid), EMBASE, CINAHL (via EBSCOhost), Web of Science, Scopus, and PsycINFO (via Ovid) was conducted to identify relevant studies. Hand-searching of reference lists of eligible articles was performed. The search strategy included keywords which were co-developed by the authors and an expert librarian and related to resilience or passion, healthcare workers, and interventions (see Appendix 1).

### 2.4 Study selection

In the first round, articles retrieved from each database were screened using Endnote software to remove the duplicates (SU). Titles and abstracts of the literature search results were then screened independently by two authors (SU, EMF) using Rayyan software. Full texts of potentially eligible articles were retrieved and assessed by two authors (SU, EMF) against eligibility criteria. Any discrepancies regarding eligibility were resolved through discussion and consensus with a third author (AEM), resulting in the final set of articles for inclusion in this review.

### 2.5 Data extraction and synthesis

Data were extracted from included studies using a standardized form, including author/s, year, country, aim, study design, method, sample, setting, characteristics of intervention, resilience and/or passion for work measurement tools used, and relevant outcomes. Data were initially extracted by SU, and accuracy and completeness of data extraction for each paper was confirmed by EMF and/or AEM. The methodological quality of included studies was assessed using the Mixed Methods Appraisal Tool version 2018 for quantitative and qualitative research designs (Hong et al., 2018). The studies that had low methodological quality assessment scores (<3 out of 5) were excluded. A summary of the methodological quality assessment of included studies is presented in Appendix 2. Findings from quantitative and qualitative studies were synthesized separately using a convergent synthesis design and then both results were combined using a narrative approach (Hong et al., 2017). Themes related to characteristics and efficacy/effectiveness of interventions were identified.

## 3 Results

### 3.1 Search results

**Fig. 1:**
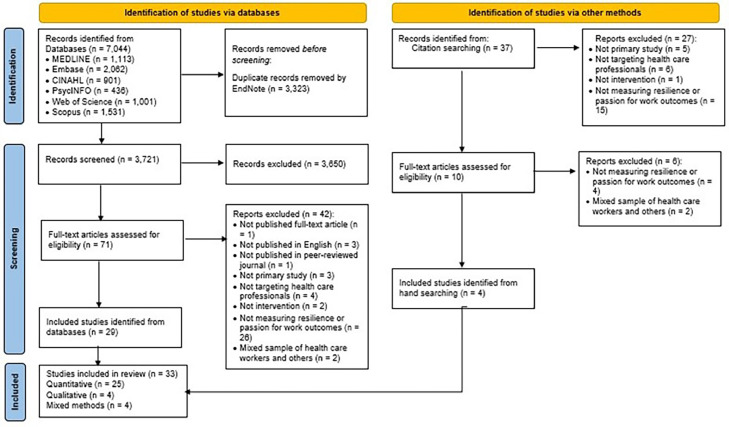
Flow diagram of the systematic review process. An adapted PRISMA 2020 flow diagram for new systematic reviews which include searches of databases, registers, and other sources (Page et al., 2021).

The PRISMA flowchart describing the review process is shown in the Fig. 1. The initial search across six databases yielded 7044 studies. Using EndNote software, 3323 studies were identified as duplicates and removed. The high number of duplicates was likely due to overlapping content and variations in citation indexing in the selected databases. Following removal of duplicates and screening by title and abstract, 71 articles met the criteria for full-text review. Of these, 29 articles were eligible for inclusion in the review (see Fig. 1). A further 37 potentially eligible studies were identified via searches of articles citing the included studies; of these, four were eligible for inclusion. Thus, a total of 33 studies met the inclusion criteria.

### 3.2 Characteristics of included studies

A summary of the characteristics of included studies is presented in Table 2. Studies were published between 2013 and 2023. Most (*n* = 25) used quantitative methods (randomised controlled trials, *n* = 11; quasi-experimental, *n* = 13; prospective cohort, *n* = 1). Four used qualitative methods and four used mixed methods. Most were conducted in developed countries (United States, *n* = 17; United Kingdom, *n* = 4, Australia, *n* = 4; Canada, *n* = 3; South Africa, *n* = 1), while only three were conducted in developing countries (China, *n* = 2; Iran, *n* = 1) and one did not provide the research setting information. Most studies (*n* = 21) recruited only nurses, nine studies recruited a mix of healthcare workers, and three recruited only physicians. Most studies were conducted in a single hospital (*n* = 22), although 11 studies were conducted in multi-site health services or research institute settings. All studies reported on interventions designed to improve resilience. No studies reported interventions to enhance passion for work. However, Duva et al. (2022) measured work engagement, where employees feel energetic and effective in their tasks and manage job demands well. While related to passion, work engagement differs and is defined as “a strong inclination towards an activity that an individual enjoys, values, and dedicates significant time and energy to” (Špehar et al., 2016 + page number).

**Table 2:** Description of included studies (*n* = 33).

References, Country	Aim	Study design, method	Sample, Setting	Characteristic of intervention	Resilience measurement	Relevant outcomes
Quantitative Randomised Controlled Trials (*n* = 11)						
Chesak et al., 2015 (USA)	Examine outcomes of the implementation of a brief Stress Management and Resiliency Training (SMART) program within a nurse orientation program	RCT pilot study	Single hospital - 40 nurses (Intervention group, *n* = 19; control group, *n* = 21)	Intervention group: A 90-minute face-to-face session, introduced model of stress and resilience integrating neuroscience and biology. Included mind-body approaches to managing stress: developing intentional attention, practicing gratitude, compassion, acceptance, forgiveness, and higher meaning. - Bi-weekly emailed handouts on each of the topics - 1-hour follow-up session to address individual questions at 4-weeks post-intervention Control group: Lecture covering topics related to stress including reality shock and work-life connectedness	CD-RISC-25; baseline, 12-weeks post-intervention	No significant intervention effect for resilience
Duva et al., 2022 (USA)	Test the effect of brief, virtual Community Resiliency Model (CRM) training to support HCW well-being, work engagement and interprofessional teamwork during the pandemic	RCT	Two large urban health systems - 253 front-line HCWs (intervention group, *n* = 126; waitlist control group, *n* = 127)	Intervention group: 1-hour virtual (online) CRM class. Included skills to increase somatic awareness in the context of self and other care, bringing attention to body sensations in the present moment to interrupt autonomic responses to stress, and cultivation of body awareness as a means of developing resilience and coping. - Delivered by certified CRM teachers - Use of ‘ichill’ app for skill reinforcement Waitlist control group: Received CRM training after final survey completed at 3 months	CD-RISC-2; baseline, 1-week and 3-months post-intervention	No significant intervention effect for resilience
Dyrbye et al., 2019 (USA)	Explore the effect of a professional coaching intervention on the well-being of physicians	RCT	Multisite trial (community-based hospitals and health care facilities) - 88 practicing physicians (intervention group, *n* = 44; control group, *n* = 44)	Intervention group: Six telephone coaching sessions (total of 3.5 h) - 1-hour initial professional coaching session (creating the relationship, assessing needs, identifying values, setting goals, creating an action plan) - 5 × 30-minute professional coaching sessions every 2 to 3 weeks over 5 months (check in, debrief strategic action, plan and set goals, design actions to incorporate into daily life, commit to next steps, and check out and summarise) - Delivered by professional coaches	CD-RISC-10; baseline and 5-months post-intervention	Intervention group had a significantly greater increase in resilience compared with control group (relative change, 4.2% vs 2.0 %; *p* = .04)
Grabbe et al., 2020 (USA)	Test the effectiveness of Community Resiliency Model (CRM) training	RCT	Two urban tertiary-care hospitals - 77 registered nurses (intervention group, *n* = 40; control group, *n* = 37)	Intervention group: 3-hour psychoeducation/sensory awareness skills training class using lecture, active engagement, discussion, demonstration, and participation. - Included practice of CRM skills (Tracking, Resourcing, Grounding, Gesturing, Shift and Stay, and Help Now!) - Delivered by certified CRM teachers - Use of “ichill” app after the class Control group: 3-hour class on nutrition/healthy eating - Use of “My Plate” app after the class	CD-RISC-10; baseline, 1 week, 3 months, and 1-year post-intervention	No significant intervention effect for resilience
Henshall et al., 2023 (UK)	Assess engagement, acceptability, and effect of Resilience Enhancement Online Training for Nurses (REsOluTionN)	Pilot RCT	Single site (mental health and community trust) - 107 nurses (intervention group, *n* = 56; waitlist control group, *n* = 51)	Intervention group: Web-based training (pre-reading, web-based facilitated sessions, mentorship support) over 4 weeks - 4 × 120-minute weekly large-group facilitated sessions covered 4 modules: 1) building hardiness and maintaining a positive outlook, 2) intellectual flexibility and emotional intelligence, 3) reflective and critical thinking, and 4) achieving life balance and enabling spirituality - 4 × 30-minute independent preparatory web-based learning on the module topics - 8 × 30–60-minute twice-weekly small group mentoring sessions (led by senior nurses)	Brief Resilience Scale; baseline and 6 weeks post-intervention	No significant intervention effect for resilience
Lin et al., 2019 (China)	Evaluate the effects of a modified mindfulness-based stress reduction (MBSR) program on stress, affect, and resilience	RCT	7 tertiary-level general hospitals - 90 nurses (intervention group, *n* = 44; waitlist control group, *n* = 46)	Intervention group: 8-week mindfulness-based group program, based on MBSR and mindfulness-based cognitive therapy (MBCT) - 8 × 2-hour weekly group sessions, including guided practice, education, and dialogues around observations of feelings, thoughts and body sensations during practice - 20 min per day, 6 days per week of formal mindfulness practice - WeChat group for sharing session PowerPoint slides, audio recordings of guided mindfulness exercises	CD-RISC-25; baseline, post-intervention, and 3-month follow-up	Significant improvement in resilience for intervention group compared to waitlist control by 3-month follow-up (*p ˂ 0*.05)
Mache et al., 2015 (Germany)	Evaluate the feasibility and effect of a psychosocial stress management and resilience training program to promote resilience and job satisfaction and decrease perceived stress	RCT	Single hospital - 85 junior physicians (intervention group, *n* = 42; waitlist control group, *n* = 43)	Intervention group: 12 × 2-hr weekly group sessions of psychosocial resilience training combined with cognitive behavioural and solution-focused counselling. - Topics include self-esteem and self-awareness, resilience, positive thoughts and emotions, cognitive behavioural training, goal setting, social support, communication, conflict handling, dealing with difficult decisions, coping with work related stress and relaxation - Delivered via psychoeducation, videos, discussions, experiential exercises, home assignments - Focus on coping strategies, support between the participants, and solutions and goals for the future - Delivered by 2 psychologists	4-item Brief Resilient Coping Scale; baseline, 3 post-intervention and 6-month follow-up	Significant improvement in resilience for intervention group compared to waitlist control at 3- and 6-months post-intervention (*p* = .01)
Mao et al., 2021 (China)	Explore the effects of emotional intelligence training on emotional intelligence, resilience, and perceived stress	RCT	Tertiary general hospital - 103 nurses (intervention group, *n* = 53; control group, *n* = 50)	Intervention group: Emotional intelligence training in two phases - Phase 1: system training phase, class lectures (2 per week for 4 weeks) used to explain emotional intelligence. Covered themes such as perception of emotions, awareness of emotions, regulation of emotions and practice - Phase 2: consolidated learning phase, weekly emotional management case discussions (60–90mins) for 44 weeks - Intervention was delivered by educators experienced in clinical psychology teaching. Control group: Daily briefings to discuss specific problems in meeting between head nurses	CD-RISC-25 measured at baseline and post- intervention	Significant increase in resilience score in the intervention group compared to the control group (*p* ˂ 0.001)
Mealer et al., 2014 (USA)	Determine the effect of a multimodal resilience training program for ICU nurses in increasing resilience	RCT	Single academic hospital, mixed ICUs - 27 nurses (intervention group, *n* = 13; control group, *n* = 14)	Intervention group: 12-week resilience training program - Starts with two-day educational workshop to introduce resilience training and the types of psychological distress experienced in the ICU. - Weekly written exposure therapy (12 × 30-minute sessions) using emailed prompts (e.g., challenges faced at work, feeling incapacitated, feeling conflicted, ruminating about sensitive topics), and feedback provided to encourage resilience-building - Mindfulness-based stress reduction practice (including body scan, sitting meditation) for 15 min weekly - Aerobic exercise (30–45 min) for at least three days per week - One event-triggered cognitive behavioural therapy session (30–60 min) with an experienced licensed clinical social worker	CD-RISC-25; baseline and 3-month post-intervention	No significant intervention effect for resilience
Slatyer et al., 2017 (Australia)	Assess the effectiveness of a brief mindful self-care and resiliency intervention in reducing burnout, secondary traumatic stress and symptoms of general psychological distress	RCT	Public teaching tertiary hospital - 91 nurses (intervention group, *n* = 65; waitlist control group, *n* = 26)	Intervention group: A full-day educational workshop comprising 4 × 1.5-hour sessions to educate about compassion fatigue, resilience and introduce participants to the mindfulness concept and basic practice. - Client manual (provided at workshop commencement) includes information about compassion fatigue and “antibodies” against compassion fatigue (self-regulation, intentionality, perceptual maturation, connection and social support, and self-care and revitalisation) - 3 × 1.75-hour weekly follow-up sessions	CD-RISC-10 measured at baseline, post-intervention, and 6-month follow-up	No significant intervention effect for resilience
Sood et al., 2011 (USA)	Assess the effect of a Stress Management and Resiliency Training (SMART) program for increasing resiliency and quality of life, and decreasing stress and anxiety	RCT	Tertiary care medical centre - 32 physicians (intervention group, *n* = 20; waitlist control group, *n* = 12)	Intervention group: A single 90-minute one-on-one SMART training to decrease stress and enhance resilience focusing on human experience, attention, and interpretation - Training in a brief structured relaxation intervention (deep breathing meditation), practice for 5–15 mins once/twice per day - Optional 30–60 min follow-up session	CD-RISC; baseline and 8 weeks post-intervention	Significant increase in resilience in the intervention group compared to the wait-list control group (*p* = .001) with a large effect size (*d* = 1.16)
Quantitative Non-Randomised Trials (*n* = 14)						
Babanataj et al., 2018 (Iran)	Determine the effect of resilience training program on occupational stress and resilience	Quasi‐experimental, single group	Single university hospital, ICUs and haemodialysis unit - 30 ICU nurses	- Group workshops, 5 sessions of between 90- and 120‐minutes duration - Covered three dimensions: resilience and the characteristics of resilient people, internal and external supportive factors, and methods to develop resilience - Methods: questions and answers, group discussion, and lectures - Delivered by a trained researcher (lecturer in faculty of nursing) under the supervision of a psychiatrist	CD-RISC-25; baseline and 2-weeks post-intervention	- Significant increase in resilience from pre- to post-intervention (*p* = .001)
Blackburn et al., 2020 (USA)	Develop an evidence-based program for addressing the concerns of burnout and secondary trauma and building on the concept of resilience in oncology healthcare providers	Quasi‐experimental, single group	- Single cancer hospital and single research institute - 164 oncology staff	- Eight-hour retreat sessions: spirituality, mindfulness, art therapy, aromatherapy, guided imagery, - 6-week private group study interaction on a social media platform - 2-hours wrap-up session focusing on a review of techniques learned and commitment to ongoing application	CD-RISC-25; baseline, immediately after intervention, and 2, 4, 6 months follow-up	- Resilience scores significantly increased pre- to post intervention (*p* = .0268)
Bonamer and Aquino-Russell, 2019 (USA)	Assess the impact of Transcendental Meditation Education program on compassion fatigue and resilience	Quasi‐experimental, single group	Single hospital - Nurses (pre-intervention, *n* = 26; post-intervention, *n* = 21)	- Introductory class, preparatory lecture, personal interview, and four consecutive days of 90–120 min of personal and group instruction taught by one of two certified Transcendental Meditation teachers - Self-practice of Transcendental Meditation (two 20-minute meditation sessions daily) - Alternating personal and group meetings every two weeks for reminder of the study	CD-RISC-25; pre- and post-intervention (4 months later)	- Resilience scores were significantly higher at 4 months compared to baseline (*p* ˂ 0.001).
Craigie et al., 2016 (Australia)	Evaluate of the effectiveness of a mindful self-care and resiliency (MSCR) intervention	Quasi‐experimental, single group	Single large teaching hospital - Nurses (pre-intervention, *n* = 25; post-intervention, *n* = 20)	- 1-day educational workshop on compassion fatigue resiliency (three 1.75-h sessions) and introduction to mindfulness (1.75-h session) - Compassion fatigue resiliency sessions included education about compassion fatigue and its causes, skills to build resiliency (5 “antibodies” against compassion fatigue: self-regulation, intentionality, perceptual maturation, connection and social support, self-care and revitalisation) - Mindfulness sessions included experiential activities, didactic discussion, brief (10–25 min) mindfulness practices - Followed immediately by a series of weekly mindfulness skills seminars conducted over a period of 4 weeks - Delivered by registered clinical psychologist	CD-RISC-25; pre- and post-intervention and 1-month follow-up	- No significant change in resilience score at post-intervention or follow-up
DeTore et al., 2022 (USA)	Examine the feasibility and acceptability of a brief online resilience-enhancement course (Resilience Training for Healthcare Workers) to increase resilience and decrease emotional distress in healthcare workers during the pandemic	Quasi-experimental, two groups	Community and specialty hospitals - HCWs (baseline *n* = 554, post-intervention *n* = 148 (intervention *n* = 38, control *n* = 110))	- Three pre-recorded videos (developed by experienced doctoral level clinicians): 1) concept of resilience and mindfulness skills (19 min), 2) enhancing cognitive flexibility via cognitive behavioural and mentalization skills (16 min) and 3) the development of self-compassion (12 min) - Included didactic information, experiential exercises, and testimonials of healthcare workers about their use of mindfulness, mentalisation, and self-compassion	2-item Brief Resilience Scale; baseline, 1-month post-intervention and 2-month follow-up	- Significant group by time interaction for improvement in resilience, sustained over the two-month follow-up in participants who took training course compared to participants who did not (*p =* .031)
Fortney et al., 2013 (USA)	Investigate the effect of a modified mindfulness-based stress reduction program on job satisfaction, quality of life, and compassion among primary care clinicians	Quasi-experimental, single group	Departments of family medicine, internal medicine and paediatrics in a university - 30 clinicians (physicians, physician assistants, nurse practitioner)	- 4-week program: 1) Mindfulness training led by mindfulness instructors (14 hrs over 3 consecutive days) – provided training in mindfulness practices (sitting, movement, speaking, listening, and compassion for self and others) and their application to practicing medicine and everyday life 2) Two x 2-hr evening follow-up sessions to guide sitting and walking mindfulness practices and encourage participants to report their experiences in a shared group setting 3) Web site to assist clinicians in bringing their mindfulness practice into their consultations with patients - Delivered by trained mindfulness instructors	14-item Resilience Scale; baseline, 1-day, 8-weeks post-intervention, 9-months follow-up	- No significant change in resilience over time.
Franco and Christie, 2021 (USA)	Evaluate the effect of the Self Compassion for Healthcare Communities (SCHC) workshop on paediatric nurses' resilience, well-being, and professional quality of life	Quasi-experimental, two groups	An urban paediatric hospital - 48 nurses (intervention group, *n* = 22; comparison group, *n* = 26)	- One-day (6 hr) adaptation of the Mindful Self-Compassion program (“Self-Compassion for Healthcare Communities”) - Six 1-hr sessions covering self-compassion, mindfulness, and well-being - Included writing activities (how do I treat a friend versus myself, writing a compassionate note to myself, reflecting on core values) and short facilitator-led practices (finding a supportive gesture, moments of mindfulness, self-compassion break) - Included a group exercise and handouts of content	Resiliency activation and resiliency decompression; pre- and post-intervention and 3-month follow-up	Significant intervention effect for resiliency decompression (*p*<.01) but no significant effect for resiliency activation (*p*=.55)
Haugland et al., 2023 (USA)	Evaluate the effect of a 3-strategy resilience bundle on resilience and stress in emergency nurses	Quasi-experimental, single group	Single hospital, emergency department - Nurses (baseline *n* = 47, week 6 *n* = 26, and week 15 *n* = 23)	Implementation of 3-element resilience bundle: 1) Mindfulness and relaxation techniques added to daily shift huddles 2) Structured debriefing led by charge nurses after a death on the unit, a highly stressful or emotional event, or at the charge nurse's discretion 3) Serenity room for relaxation and restoration during work shifts	CD-RISC-10; baseline, follow-up at 6–15 weeks post-baseline	Significant increase in mean rank for resilience from baseline to 6-weeks post-intervention (*p* = .03)
Kelly et al., 2021 (South Africa)	Evaluate the effect of a self-directed e-learning intervention on resilience-building behaviours, resilience and wellbeing	Quasi-experimental, single group	Public and private hospitals - 474 HCWs	- Online 5-module e-learning course targeting resilience and well-being during COVID-19 - Content focused on improving HCW's knowledge of stress in the workplace, how stress has been exacerbated by COVID-19, and improving ability to manage stress, trauma and distress using practical strategies - Asynchronous learning model; content delivered through narrated presentation slides and additional resources (e.g., journal articles, websites) - Interactive, including quizzes, discussion boards, practical activities (e.g., self-assessments of burnout and psychological well-being, practicing mindfulness and meditation, and developing a self-care plan) - Self-care plans included practices across five categories: physical, mild/spiritual, relationships, emotions, and work - Participants completed online modules within 1 month	CD-RISC-10; pre- and post-intervention	Significant increases in resilience (*p* < .001) and resilience-building behaviours (*p* < .001)
Magtibay et al., 2017 (USA)	Assess the efficacy of the Stress Management and Resiliency Training (SMART) program to decrease stress and burnout among nurses	Quasi-experimental, single group	Single teaching hospital - Nurses (week 8 *n* = 20, week 12 *n* = 15, week 16 *n* = 2, and week 24 *n* = 1)	- SMART program offered via blended learning (participant choice of web-based content, independent reading, facilitated discussions, or a combination of these) - Web-based: 12 modules of independent learning including pre-module and post-module self-assessment, brief videos to introduce the topic, reading assignments, and exercises to apply content - Facilitated discussions: 4 sessions (weeks 8 and 12 in-person; weeks 16 and 20 via telephone) to problem solve and answer questions	CD-RISC-2; baseline and weeks 8, 12, and 24 post-baseline	Significant increases in resilience level from baseline to weeks 12 (*p* = .048) and 24 (*p* = .004)
Mintz-Binder et al., 2021 (USA)	Investigate the feasibility of increasing nurse resiliency through a toolkit of evidence-based stress-reducing interventions	Quasi-experimental, single group	- Four affiliated urban and suburban hospitals - Nurses (baseline *n* = 148, week 4 and 6 *n* = 77)	Nurses were offered a variety of resilience-building strategies: lavender aromatherapy, adult colouring books with pencils, deep breathing exercise with app, relaxation through guided meditation with app, gaming apps (Bejeweled, Tetris), mind activity book (Sudoku, crossword puzzles, word search games)	CD-RISC-10; pre- and post-intervention	Significant increases in resilience level at follow-up (*p* ˂ 0.02)
Muir et al., 2022 (USA)	Describe the feasibility, accessibility, and effectiveness of a mobile well-being intervention, “Room to Reflect (R2R)”, on nurse staff and nurse manager resilience	Quasi-experimental, single group	Single academic medical centre - Nurses (baseline *n* = 97, post-intervention *n* = 57)	Two months’ access to R2R, which included two intervention elements: 1) Mobile toolbox (sound machine, virtual reality headset, audio/video resources accessible using personal mobile devices) providing access to 5 categories of offerings: restorative movement with yoga and stretching, soothing sounds, inspiration poems and quotes, meditation, and virtual reality 2) Pocket Guide of mindful restoration practices to use during provision of clinical care	CD-RISC-10; pre- and post-intervention	Significant increase in resilience for nurse managers (*p* < .05) but not staff nurses (*p* = .19)
Rushton et al., 2021 (USA)	Examine the impact of an experiential educational curriculum to enhance nurses’ skills in mindfulness, resilience, confidence, and competence to confront ethical challenges in clinical practice	Quasi-experimental, two groups	Two hospitals - Nurses (pre-intervention *n* = 192, post-intervention *n* = 164)	Curriculum of the Mindful Ethical Practice and Resilience Academy: Cultivating moral resilience through knowledge, skills and practice in self-regulation, mindfulness, moral sensitivity, discernment and action, targeted communication skills to enhance moral efficacy, and methods for translating new skills into practice 1) 6 experiential sessions (4 h each) of face-to-face, interactive training based on a variety of educational and evaluative methods (didactic experiential practices, role play, video review, mindfulness practice, group activities, high-fidelity simulation and facilitated reflective debriefing 2) 10 min of daily technology enabled mindfulness (breathing, loving-kindness, difficult emotions, letting go) and reflective practice	6-item Brief Resilience Scale; pre- and post-intervention	Resilience significantly improved after participating intervention (*p* < .001)
Yi-Frazier et al., 2022 (Not mentioned)	Assess the feasibility, acceptability, and preliminary outcomes of a skills-based coaching program designed to reduce stress and build resilience	Pilot cohort study, mixed methods analysis, survey and open-end questions	- A children's hospital and the affiliated university medical - 81 HCWs were patient-facing roles (ie, nurses, physicians, allied health professionals)	- 6-session group-based curriculum administered by 2 trained promoting resilience in stress management coaches on a video conferencing platform. - The program included 6weekly 1-hour sessions, covering (1) science of resilience, (2) stress management, (3) goal setting, (4) cognitive reframing, (5) meaning making, and (6) coming together and moving forward with resilience	CD-RISC-10; pre- and post-intervention	- Resilience among HCWs who were patient-facing role significantly increased after the program (*p* = .004)
Mixed Methods (*n* = 4)						
Crandall et al., 2022 (Canada)	Explore the effects of Mindful Self-Compassion (MSC)training on nephrology nurses’ self-reported levels of self-compassion, resilience, and burnout.	Mixed methods, survey and focus group	- Single academic hospital - 8 nurses completed survey three points - 12 nurses participated in focus group	- 8-week course to cultivate the skill of self-compassion (eight 3-hour sessions and one optional 4-hour retreat) - Online access and/or a CD containing the three Core Mindful Self-Compassion Meditations (Affectionate Breathing, Loving-Kindness for ourselves, Giving and Receiving Compassion) and the Self-Compassion Break to assist with home meditation practice	CD-RISC-25; pre, immediately post-intervention, and 3 months follow-up	- Significant increases in resilience scores between the pre-and immediately post-intervention and sustained three months later (*p* ˂ 0.001) (pre mean =55.75 SD 11.65, post training mean=74.5 SD13.5- and 3-month post training mean=73.88 SD12.86 - Intervention enhanced resilience, created a community of support, increased self-awareness through mindfulness & self-compassion techniques. This mastery of MSC was seen as increasing participants’ ability to cope with day-to-day challenges
Delaney, 2018 (Ireland)	Assess the effect of Mindful Self-Compassion (MSC)program in relation to nurses’ compassion fatigue and resilience	Pilot Mixed methods, survey and focus group	- Single hospital - 13 nurses working in cancer, cardiology, maternity, midwifery, intensive care and urology	- 8-week training teaches core principles including Mindfulness Meditation, Loving Kindness Meditation, and Compassion Meditation - Attend two-and-a-half hour training session each week and participated in a half-day retreat. - Practice daily and received four practice CDs of formal practices and informal practices	CD-RISC-25; pre- and post-intervention	- Resilience score increased from pre to post intervention (pre mean = 67.61, post mean = 80.30) - Intervention improved positive mental states, coping skills, acceptance and mindful awareness. - Intervention reduced stress, staff reported they were less self-critical, and had difficulty practicing MSC skills.
Henshall et al., 2020 (England)	Implement and evaluate a work-based personal resilience enhancement intervention for forensic nurses.	Mixed methods, surveys and interviews	- A mental health and community, the National Health Service forensic inpatient wards - 26 nurse mentees participated surveys - 12 nurse mentees and 12 nurse mentors participated interview	- 6 full-day face to face sessions over 12 weeks - Program consisted of a variety of workshops and tackled areas such as building hardiness, maintaining a positive outlook, achieving work-life balance, reflective and critical thinking, and enabling spirituality	1-item in a 5 Likert scale (ranging from low (1) to high (5)) evaluated aspects of nurses’ levels of resilience-pre- and post-programme	- Significant increases in personal resilience level at post-intervention among mentees (Pre mean= 3.42 SD 0.70 Post mean =4.12 SD 0.60) (*p* = .0004) - Mentees indicated that the programme had impact on personal resilience, self-awareness, confidence, and professional relationships
Kim et al., 2022 (Canada)	Explore the effects of an online mindfulness program on resiliency in health care workers during the pandemic	- Mixed-methods, survey and semi-structured interviews	- Single psychiatric hospital - 130 HCWs completed pre-, post-, and follow-up surveys - 10 HCWs participated in interviews	- 4-week online Mindfulness Ambassador Program via Zoom in fostering resiliency - Each mindfulness session ran once a week for 30 min, with on certified mindfulness ambassador program facilitator assigned to each group (max 20)	12-item McBride Resilience Questionnaire-pre, post, and 1-month follow-up	- Resilience score significantly increased after the program (*p* ˂ 0.05) compared to the baseline, maintaining the effect after one month (p ˂ 0.01) - Intervention had a positive effect on empathy and resiliency by providing time and space for practicing self-care and teaching to embody mindfulness.
Qualitative Studies (*n* = 4)						
Esplen et al., 2022 (Canada)	Examine the effect of a Mindfulness based stress reduction, Self-Compassion and wellness and support training on nurses’ compassion fatigue and resilience and experiences of the effect of the training.	Qualitative description, open-ended questions and reports of participant plans, content analysis	- Multisite (cancer centres, community care, palliative care, and general hospital units) - 189 HCWs (93 % nurses)	- A 6-week continuing education program consisting of weekly 1.5 h small-group video conferencing sessions with case-based learning discussions - Program content included personal, organization and team-related risk and protective factors (compassion fatigue, grief models, and strategies to mitigate against compassion fatigue)	–	- Continuing education to enhance knowledge and confidence around grief and loss, self-monitoring, knowledge of self, and team-based strategies including sharing experiences in a Community of Practice help to support resilience
Foster et al., 2018 (Australia)	Explore the perspectives of mental health nurses participating in the Promoting Adult Resilience program	Exploratory qualitative, open-ended responses, semi- structured interviews and focus groups, thematic analysis	- A large metropolitan public mental health service - 29 nurses participated in total with 24 completing open-ended written data - 4 nurses participated in interviews – 8 nurses participated in focus groups	- Face-to-face group discussion and individual activities over 7 weeks with one module/week - Contents; recognizing strengths and understanding resilience, understanding and handling stress, challenging and changing negative self-talk, drawing strength from hardship, supporting positive relationships, managing conflict, generating solutions for well-being and bringing it all together	–	- Perceived positive outcomes after the programme; being confronted by adversity, reinforcing understandings of resilience, strengthening resilience, and applying resilience skills at work including emotional self- regulation, cognitive behavioural responses to stress (positive self-talk, detaching from emotions & managing responses, problem solving, empathy)
McDonald et al., 2013 (Australia)	Explore the perspectives of nurses and midwives participating in the work-based, educational interventions that focus on personal resilience	Exploratory qualitative, semi- structured interviews, thematic analysis	- A women's and children's health service in a large, tertiary referral hospital - 14 nurses and midwives	- Six resilience workshops and a mentoring programme conducted over a 6-month period over a whole day each month, onsite at the hospital - Characteristics associated with resilience: positive and nurturing relationships and networks, mentoring, positive outlook, hardiness, intellectual flexibility, emotional intelligence, life balance, spirituality, reflection, and critical thinking	–	-Perceived personal gains from resilience workshops including experiential learning that fostered sharing of experiences, support and trust, creative self-expression and improved self-awareness, learning new ideas and resilience strategies. Professional gains included increased assertiveness at work, improved workplace relationships and communication and a greater understanding of self-care and personal resilience practices.
Potter et al., 2015 (USA)	Describes the facilitators’ perceptions of the effects of participating in a compassion fatigue resiliency training program	Phenomenology, semi- structured interviews, narrative analysis	- A large tertiary academic medical centre - 25 HCWs including nurses, pastoral care, social work, psychologist, physician assistant and human resource and cultural diversity staff	- A 2-day facilitator training program using didactic learning, small group activities and discussions - Components: self-regulation, intentionality, perceptual maturation, connection, and self-care	–	- HCWs perceived improvements in application of resiliency strategies and techniques to daily lives including self-regulation, intentionality, self-care, connection, and perceptual maturation.

Note: CD-RISC = Connor-Davidson resilience scale; RCT = Randomized controlled trials; HCW = Healthcare workers; CRM = Community Resiliency Model; ICU = Intensive care unit.

### 3.3 Characteristics of interventions

#### 3.3.1 Content and delivery

Interventions varied in format, intensity, duration, and content. Many interventions were designed with multiple components which included, for example, mindfulness training, psychoeducation, stress management techniques and/or professional coaching.

Mindfulness-based interventions were most frequently reported and tested in 17 studies (Blackburn et al., 2020; Craigie et al., 2016; Crandall et al., 2022; Delaney, 2018; DeTore et al., 2022; Esplen et al., 2022; Fortney et al., 2013; Franco and Christie, 2021; Haugland et al., 2023; Kelly et al., 2021; Kim et al., 2022; Lin et al., 2019; Mealer et al., 2014; Mintz-Binder et al., 2021; Muir et al., 2022; Rushton et al., 2021; Slatyer et al., 2017). These interventions typically aimed to cultivate present-moment awareness, mindful self-care, self-regulation of thoughts and emotions, self-compassion, and stress reduction. Commonly reported mindfulness practices included deep breathing exercises, body scans, meditation, art therapy, aromatherapy, yoga and stretching, gaming and technology enabled mindfulness (breathing, loving-kindness, difficult emotions, letting go). Mindfulness interventions were typically delivered through one-to-one, or group guided sessions led by trained facilitators, or through self-directed practices using handouts, audio recordings, mobile applications, or social media platform or web-based training.

The Community Resiliency Model (CRM) training uses the concept of sensory awareness, distinguishing it from earlier mindfulness approaches, to promote the well-being of healthcare workers (Duva et al., 2022; Grabbe et al., 2020). This model focuses on somatic awareness in the context of self-care and care for others, emphasizing attention to body sensations in the present moment to interrupt autonomic stress responses and cultivating body awareness as a means of developing resilience and coping (Duva et al., 2022).

Psychoeducation workshops were also commonly reported components of resilience interventions and tested in 10 studies. Psychoeducational interventions typically involved providing information and increasing participants’ knowledge about the Community Resiliency Model (Duva et al., 2022; Grabbe et al., 2020), resilience enhancement (Babanataj et al., 2018; Foster et al., 2018; Henshall et al., 2020, 2023), emotional intelligence (Mao et al., 2021; McDonald et al., 2013), or psychological distress (Mealer et al., 2014; Potter et al., 2015). Topics covered in psychoeducation sessions included understanding the concept of resilience (characteristics of resilience, internal and external supportive factors, and methods to develop resilience), understanding and handling stress, identifying personal strengths, building hardiness, maintaining a positive outlook, achieving work-life balance, reflective and critical thinking, and enabling spirituality and generating solutions for well-being. Psychoeducation sessions were often delivered through workshops which included a combination of didactic and active learning activities including lectures, questions and answer sessions, group discussion, and mentoring activities. However, not all interventions were delivered face-to-face; web-based training and mobile applications were used to provide psychoeducation intervention in several studies (Duva et al., 2022; Grabbe et al., 2020; Henshall et al., 2023).

Stress management techniques were frequently included as components of resilience interventions and were tested in five studies. Many combined psychosocial stress management and resiliency training to simultaneously decrease stress and enhance resilience (Chesak et al., 2015; Mache et al., 2015; Magtibay et al., 2017; Sood et al., 2011; Yi-Frazier et al., 2022). Stress management interventions included components that addressed mind-body approaches to managing stress, self-esteem, self-awareness, positive thoughts and emotions, cognitive behavioral training, goal setting, social support, communication, conflict management, dealing with difficult decisions, solution-focused counselling, coping with work related stress and relaxation. Most stress management approaches were delivered through on-site workshops using lectures, videos, discussions, experiential exercises, and home assignments, while three studies added self-directed e-learning to improve knowledge of stress management (Kelly et al., 2021; Magtibay et al., 2017; Yi-Frazier et al., 2022). Moreover, follow-up sessions were held in-person or via telephone to problem solve and answer participants’ questions (Chesak et al., 2015; Magtibay et al., 2017; Sood et al., 2011).

Professional coaching was included as a component of resilience interventions reported in three studies and delivered in varied formats (Dyrbye et al., 2019; Kim et al., 2022; Yi-Frazier et al., 2022). Coaching sessions focused on strategic action, stress management, planning and setting goals, cognitive reframing, and moving forward with resilience. Coaching sessions were delivered by trained professionals through individual telephone-based sessions (Dyrbye et al., 2019) or group-based coaching sessions delivered via video conferencing or web-based platforms (Kim et al., 2022; Yi-Frazier et al., 2022).

#### 3.3.2 Duration of intervention

Included studies reported a wide range of intervention intensities and durations, highlighting the diversity in approaches to promoting resilience among healthcare works. The interventions varied in duration from 2 weeks to 2 years, with a median duration of 8 weeks. (Blackburn et al., 2020; Chesak et al., 2015; Delaney, 2018; Duva et al., 2022; Fortney et al., 2013; Franco and Christie, 2021; Grabbe et al., 2020; Haugland et al., 2023; Lin et al., 2019; Mealer et al., 2014; Rushton et al., 2021; Slatyer et al., 2017; Sood et al., 2011).

#### 3.3.3 Outcome measures

All studies reported resilience as an outcome, but none reported a passion for work as an outcome (Table 2). Of the 29 studies that included a quantitative measure of resilience, the Connor-Davidson Resilience Scale (CD-RISC) was most frequently used (21/29) with different versions. Of these, 10 studies used a 25-item version with nurses and oncology staff (Babanataj et al., 2018; Blackburn et al., 2020; Bonamer and Aquino-Russell, 2019; Chesak et al., 2015; Craigie et al., 2016; Crandall et al., 2022; Delaney, 2018; Lin et al., 2019; Mao et al., 2021; Mealer et al., 2014); eight used the 10-item version with physicians, nurses and a mix of healthcare workers (Dyrbye et al., 2019; Grabbe et al., 2020; Slatyer et al., 2017; Haugland et al., 2023; Kelly et al., 2021; Mintz-Binder et al., 2021; Muir et al., 2022; Yi-Frazier et al., 2022); two used a two-item version with front-line healthcare workers and nurses (Duva et al., 2022; Magtibay et al., 2017); and one did not report the version of the CD-RISC that was used (Sood et al., 2011).

Other measures of resilience included the Brief Resilience Scale (DeTore et al., 2022; Henshall et al., 2023; Rushton et al., 2021), Brief Resilient Coping Scale (Mache et al., 2015), Resilience Scale (Fortney et al., 2013), Resiliency Activation and Resiliency Decompression Scale (Franco and Christie, 2021), and McBride Resilience Questionnaire (Kim et al., 2022). In one study, nurses' levels of resilience were assessed using a single-item measure, employing a 5-point Likert scale ranging from low (1) to high (5) without a report of measurement development process and validity (Henshall et al., 2020). All the 29 experimental studies performed a pre- and post-intervention assessment of resilience, 12 conducted additional follow-up assessment (ranging from 1 to 9 months post-intervention), and three also assessed resilience at specific timepoints during implementation of the intervention. Four qualitative studies explored participants’ perceptions of the effectiveness of the intervention using individual interviews, focus groups, open-ended questions, and written reports from participants (Esplen et al., 2022; Foster et al., 2018; McDonald et al., 2013; Potter et al., 2015).

### 3.4 Effectiveness of interventions

#### 3.4.1 Outcomes of quantitative evidence

Among the 29 studies reporting quantitative data on change in resilience from pre- to post-intervention, most (21/29) reported statistically significant increases in resilience (Babanataj et al., 2018; Blackburn et al., 2020; Bonamer and Aquino-Russell, 2019; Crandall et al., 2022; Delaney, 2018; DeTore et al., 2022; Dyrbye et al., 2019; Franco and Christie, 2021; Haugland et al., 2023; Henshall et al., 2020; Kelly et al., 2021; Lin et al., 2019; Kim et al., 2022; Mache et al., 2015; Magtibay et al., 2017; Mao et al., 2021; Mintz-Binder et al., 2021; Muir et al., 2022; Rushton et al., 2021; Sood et al., 2011; Yi-Frazier et al., 2022).

Most studies that employed mindfulness as one of a combination of intervention components reported statistically significant positive changes in resilience (Blackburn et al., 2020; Crandall et al., 2022; Delaney, 2018; DeTore et al., 2022; Franco and Christie, 2021; Haugland et al., 2023; Kim et al., 2022; Lin et al., 2019; Muir et al., 2022; Rushton et al., 2021), while only two interventions that included a mindfulness component resulted in no significant intervention effect (Fortney et al., 2013). Among the six studies investigating the effect of combined stress management and resiliency training, almost all reported significant increases in resilience level from baseline to post-intervention (Mache et al., 2015; Magtibay et al., 2017; Mintz-Binder et al., 2021; Sood et al., 2011; Yi-Frazier et al., 2022), and only one reported no significant intervention effect for resilience (Chesak et al., 2015). In contrast, the two studies evaluating a mindful self-care and resiliency intervention (Craigie et al., 2016; Slatyer et al., 2017), as well as the two studies examining an intervention based on the community resiliency model (Duva et al., 2022; Grabbe et al., 2020), did not yield significant effects on resilience.

Of the fifteen studies that also measured resilience at follow-up (from 1 to 9 months post-intervention), 11 demonstrated sustained intervention effects (Blackburn et al., 2020; Crandall et al., 2022; DeTore et al., 2022; Franco and Christie, 2021; Haugland et al., 2023; Kim et al., 2022; Lin et al., 2019; Mache et al., 2015; Magtibay et al., 2017; Mintz-Binder et al., 2021; Sood et al., 2011) whereas four reported no significant improvement in resilience scores at follow-up (Craigie et al., 2016; Fortney et al., 2013; Grabbe et al., 2020; Slatyer et al., 2017). Among the 17 mindfulness-based interventions, eight demonstrated the ability to sustain efficacy over time, whereas three out of five stress management interventions exhibited sustained efficacy at follow-up. Notably, no sustained efficacy from interventions were reported for psychological workshops or professional coaching.

#### 3.4.2 Outcomes of qualitative evidence

The key findings from qualitative data, comprising four mixed-method investigations and four qualitative studies, provided insight into perceived enhancements in resilience and other positive outcomes among healthcare workers as documented in Table 2.

Two studies evaluated a mindful self-compassion program using a mixed-methods approach with nurses (Crandall et al., 2022; Delaney, 2018). During the focus group, nurses perceived that learning to use mindfulness and self-compassion techniques enhanced their resilience, self-awareness, positive mental states and coping skills, and reduced stress. Nevertheless, participants also articulated the difficulty of engaging in mindfulness and self-compassion practices due to experiencing drowsiness and feelings of resistance to practising (Delaney, 2018).

One mixed-methods study and one qualitative study tested work-based educational interventions to enhance personal resilience among nurses and midwives (Henshall et al., 2020; McDonald et al., 2013). Each conducted face-to-face multi-modal workshops over a 12-week and 6-month period, respectively. Findings from interviews indicated that participants perceived gains in experiential learning, personal resilience practices, self-awareness, self-care, confidence, creative self-expression, workplace relationships and communication.

One mixed-method study and one qualitative study delivered a mindfulness program to foster resiliency among healthcare workers (Esplen et al., 2022; Kim et al., 2022). Participants emphasized that practicing self-care and teaching strategies that helped them embody mindfulness improved their resilience and empathy (Kim et al., 2022). In their evaluations of the workshop via open-ended questions, participants recommended continuing education and sharing experiences in a community of practice may help to support resilience (Esplen et al., 2022).

One qualitative study explored the effect of a program promoting resilience among nurses (Foster et al., 2018). Participants perceived positive effects of the training, noting enhancements in their knowledge regarding confronting adversity, reinforcement of their understanding of resilience, and strengthening of resilience skills, which they applied in their workplace.

One qualitative study reported on the effect of a compassion fatigue resiliency training program targeting healthcare workers (Potter et al., 2015). Facilitators delivered a two-day workshop with didactic learning, small group activities and discussions. In the post-workshop evaluation interviews, participants reported applying resiliency strategies and techniques to their daily lives.

## 4 Discussion

This mixed-methods systematic review presented a comprehensive exploration of interventions from quantitative, qualitative, and mixed-method studies aimed at promoting resilience among healthcare professionals within health settings. Overall, evidence suggests that multi-component interventions incorporating psychoeducation, mindfulness, and/or stress management components can be effective in improving resilience among healthcare workers. Indeed, most studies reporting quantitative data and all studies reporting qualitative data indicated positive intervention effects for resilience. In contrast, no studies reporting interventions to increase passion for work were found, and the complete lack of published research in this area represents an important gap in knowledge that will need to be addressed.

One of the notable findings of this review is the remarkable diversity in content, mode of delivery, and intensity and duration of interventions designed to foster resilience among healthcare workers. Most studies used multi-component interventions to enhance resilience. This finding aligns with the conclusions of a prior systematic review and meta-analysis by Joyce et al. (2018), which noted significant variability in the types of resilience training offered, albeit predominantly encompassing a blend of psychoeducation, mindfulness practices, cognitive skills development, self-compassion techniques, gratitude exercises, emotional regulation training, relaxation methods, and goal-setting strategies. However, those authors highlighted the effectiveness of resilience interventions combining cognitive behavioural therapy-based approaches with mindfulness techniques in enhancing individual resilience among the adult population (Joyce et al., 2018). Likewise, results of previous systematic reviews indicated that resilience interventions administered to healthcare professionals exhibited considerable variability concerning content, duration, delivery methods, evaluation modalities, and frequency of assessments (Cleary et al., 2018; Leppin et al., 2014).

Studies which used mindful self-care resiliency intervention and community resiliency model reported no significant improvements in resilience scores. Notably, the intervention format in these studies only comprised lectures of psychoeducation workshop, followed by participants' independent study and practice sessions, differing from other studies, which could potentially impact the efficacy of the interventions. This result is consistent with the findings of a systematic review by Cleary et al. (2018), which revealed that not all interventions effectively bolstered resilience, with results displaying variability among studies. Similarly, a systematic review and meta-analysis by Leppin et al. (2014) highlighted variability in format and theoretical underpinnings among resilience interventions, indicating a modest impact on enhancing resilience and other mental health outcomes. They further concluded that a lack of clarity persists regarding the essential components defining a resilience training program, characterized by diverse operationalizations and a dearth of common theoretical or scientific specificity. It is evident that these factors could impact efficacy, highlighting the necessity for training program frameworks to organize the various operational approaches used in intervention design. Consistent with the systematic review by Leung et al. (2022), resilience interventions often yield insignificant improvements due to various barriers, challenges, and variability in intervention design. However, more rigorous and standardized research in this area may enhance resilience more effectively.

The trend of resilience interventions for healthcare workers has grown rapidly, particularly in response to the stressors intensified by the COVID-19 pandemic, as evidenced by the large number of studies conducted (Blackburn et al., 2020; Crandall et al., 2022; DeTore et al., 2022; Duva et al., 2022; Esplen et al., 2022; Franco and Christie, 2021; Haugland et al., 2023; Henshall et al., 2020; Henshall et al., 2023; Kelly et al., 2021; Kim et al., 2022; Mao et al., 2021; Mintz-Binder et al., 2021; Muir et al., 2022; Rushton et al., 2021; Yi-Frazier et al., 2022). Various strategies have emerged, not only focusing on enhancing psychological resilience but also promoting other outcomes such as professional quality of life, well-being, confidence, and competence in confronting ethical challenges in clinical practice. Additionally, these interventions aim to reduce emotional distress, burnout, stress, and compassion fatigue among healthcare workers during the pandemic. Notably, fostering resilience has proven crucial in protecting healthcare workers facing crisis events from psychological distress.

Our review revealed that most studies utilized the CD-RISC for assessing resilience, yet there was considerable variation in the versions of CD-RISC used. Furthermore, some studies opted for alternative assessment instruments, but did not provide details regarding the validity of these instruments. Consistent with previous systematic reviews there have been observed discrepancies among the studies included, particularly regarding the assessment tools employed for measuring resilience (Chmitorz et al., 2018; Leppin et al., 2014). Furthermore, these reviews emphasized the lack of a universally accepted gold standard method for evaluation or measurement in this context. Similarly, Cleary and colleagues (2018) emphasised not all studies used standard instruments to measure resilience among health professionals. Inconsistencies in selected instruments may be due to differences in the definitions or concept of a resiliency training program used across studies. Results of the present review could guide future research endeavors aimed at establishing standardized resilience measurements grounded in theoretical resilience frameworks and better define the resilience construct relevant to health care settings.

In summary, results of this mixed-methods systematic review indicate that interventions employing a multi-component approach to enhancing resilience among healthcare professionals may yield positive outcomes for this population. Nonetheless, there remains a lack of consistency in measurement approaches and theoretical frameworks informing intervention design and outcomes. Most included studies had small samples. Variability in sample size across studies can significantly contribute to an overestimation of effect size, poor replicability, and limited generalizability of findings to broader populations (Barch, 2023). Further research is needed to develop a resilience training program framework that may help to organize intervention designs and outcome measures. No studies reported on an intervention to increase passion for work in a health setting. Thus, future research on boosting passion for work among health care professionals is needed.

## 5 Limitations

Several limitations of this systematic review should be noted. First, the heterogeneity of intervention designs and outcome measures across studies limits the ability to make direct comparisons and draw definitive conclusions. Second, the reliance on self-report measures in quantitative studies may introduce response bias and social desirability effects. Additionally, most studies were conducted in high-income countries, potentially limiting the generalizability of findings to similar settings and populations. Lastly, a meta-analysis could not be performed due to the heterogeneity of studies, precluding an estimate of overall effect size and identification of standard errors (Lin et al., 2017).

## 6 Conclusion and implications for future research

This systematic review provides valuable insights into the nature and efficacy/effectiveness of resilience interventions among healthcare workers. Although there was a variation of intervention contents and delivery formats, results suggest that a multi-component approach may be more effect to promote resilience. The standard measurement to assess resilience remain required due to used varied instrument across studies and not all studies used standard instrument. Future research should focus on establishing intervention efficacy with healthcare workers in diverse cultural and social contexts, particularly given the high levels of occupational stress experienced by healthcare workers in low- and middle-income countries. Further research also is needed to clarify the universal definition of resilience, constructs or framework of resilience building interventions and resilience outcomes measurement specific in health setting. The studies aimed to compare the effectiveness of different intervention design are needed. In addition, future research developing and investigating interventions related to build passion for work among health professionals is needed.

## Role of the funding source

The authors would like to acknowledge funding support of the Fundamental Fund of Khon Kaen University, has received funding support from the National Science, Research and Innovation Fund (NSRF) (SU, FM-FF67-001).

## CRediT authorship contribution statement

**Supan Unjai:** Writing – review & editing, Writing – original draft, Validation, Software, Project administration, Methodology, Funding acquisition, Formal analysis, Data curation, Conceptualization. **Elizabeth M. Forster:** Writing – review & editing, Validation, Supervision, Software, Methodology, Formal analysis, Data curation, Conceptualization. **Amy E. Mitchell:** Writing – review & editing, Validation, Supervision, Methodology, Formal analysis, Data curation, Conceptualization. **Debra K. Creedy:** Writing – review & editing, Validation, Supervision, Methodology, Investigation, Formal analysis, Data curation, Conceptualization.

## Declaration of competing interest

The authors declare that they have no known competing commercial interests or personal relationships that could have appeared to influence the work reported in this study.

## Appendix Supplementary materials

Image, application 1

Image, application 2

Image, application 3

Image, application 4
