# Supplementary material for: Interventions to promote resilience and passion for work in health settings: A mixed-methods systematic review
Source: Int J Nurs Stud Adv. 2024 Sep 21;7:100242. doi: 10.1016/j.ijnsa.2024.100242 (PMC11460621; doi:10.1016/j.ijnsa.2024.100242)
Supplement: Supplementary file 1 [file mmc1.docx]

**Supplementary online material (Appendix 1)**

**Search strategies and the search results**

MedLine

| **#** | **Searches** | **Results** |
| --- | --- | --- |
| 1 | exp Adaptation, Psychological/ or exp Resilience, Psychological/ or exp Social Adjustment/ | 163620 |
| 2 | exp Job Satisfaction/ or exp Employment/ | 121134 |
| 3 | 1 and 2 | 5228 |
| 4 | ((Work* or Occupation or Employ* or Job or Career) adj5 (passion* or resilien* or adapta* or cope* or coping or hardiness or motivat*)).ti,ab. | 13825 |
| 5 | 3 or 4 | 18256 |
| 6 | ((Improv* or Enhanc* or Build* or Increas* or Develop* or Promot*) adj5 (Intervention* or Program* or Train* or Behaviour or Behavior or Strateg*)).ti,ab. | 623919 |
| 7 | 5 and 6 | 2442 |
| 8 | exp Health Personnel/ or exp Health Occupations/ | 2285319 |
| 9 | (Nurs* or Doctor* or Physician* or "Health Care Provider*" or "Health Professional*" or Clinician* or "Health Practitioner*" or "Healthcare Worker*" or "Health Care Worker*" or paramedic* or "emergency medical dispatcher").ti,ab. | 1225104 |
| 10 | 8 or 9 | 3013709 |
| 11 | 7 and 10 | 1286 |
| 12 | limit 11 to yr="2003 -Current" | 1174 |
| 13 | ("Systematic review" or "Systematic literature review" or "Scoping review" or "Meta-anly*" or "Integrative review" or "Integrative literature review" or "Narrative review" or "Narrative literature review").ti. | 194322 |
| 14 | 12 not 13 | 1114 |
| 14 | 14 and "Journal Article" [Publication Type] | 1113 |

PsycInfo

| **#** | **Searches** | **Results** |
| --- | --- | --- |
| 1 | exp Resilience, Psychological/ or exp Social Adjustment/ | 30187 |
| 2 | exp Job Satisfaction/ or exp Employment/ | 52222 |
| 3 | 1 and 2 | 451 |
| 4 | ((Work* or Occupation or Employ* or Job or Career) adj5 (passion* or resilien* or adapta* or cope* or coping or hardiness or motivat*)).ti,ab. | 23923 |
| 5 | 3 or 4 | 24232 |
| 6 | ((Improv* or Enhanc* or Build* or Increas* or Develop* or Promot*) adj5 (Intervention* or Program* or Train* or Behaviour or Behavior or Strateg*)).ti,ab. | 325666 |
| 7 | 5 and 6 | 2837 |
| 8 | exp Health Personnel/ | 186904 |
| 9 | (Nurs* or Doctor* or Physician* or "Health Care Provider*" or "Health Professional*" or Clinician* or "Health Practitioner*" or "Healthcare Worker*" or "Health Care Worker*" or paramedic* or "emergency medical dispatcher").ti,ab. | 344636 |
| 10 | 8 or 9 | 439565 |
| 11 | 7 and 10 | 510 |
| 12 | limit 11 to yr="2003 -Current" | 456 |
| 13 | ("Systematic review" or "Systematic literature review" or "Scoping review" or "Meta-anly*" or "Integrative review" or "Integrative literature review" or "Narrative review" or "Narrative literature review").ti. | 39152 |
| 14 | 12 not 13 | 454 |
| 15 | 14 and "Peer Reviewed Journal" [Publication Type] | 436 |

Embase

| **#** | **Searches** | **Results** |
| --- | --- | --- |
| 1 | 'coping behavior'/exp OR 'resilience'/exp OR 'social adaptation'/exp OR 'hardiness'/exp | 222,972 |
| 2 | 'job satisfaction'/exp OR 'employment'/exp OR 'employment status'/exp | 156,166 |
| 3 | #1 AND #2 | 40,732 |
| 4 | ((work* OR occupation OR employ* OR job OR career) NEAR/5 (passion* OR resilien* OR adapta* OR cope* OR coping OR hardiness OR motivat*)):ti,ab | 20,554 |
| 5 | #3 OR #4 | 59,392 |
| 6 | (('improv' OR enhanc* OR build* OR increas* OR develop* OR promot*) NEAR/5 (intervention* OR program* OR train* OR behaviour OR behavior OR strateg*)):ti,ab | 705,381 |
| 7 | #5 AND #6 | 5,155 |
| 8 | 'health personnel'/exp OR 'health occupations'/exp | 1,952,992 |
| 9 | nurs* OR doctor* OR physician* OR 'health care provider*' OR 'health professional*' OR clinician* OR 'health practitioner*' OR 'healthcare worker*' OR 'health care worker*' OR paramedic* OR 'emergency medical dispatcher' | 3,081,706 |
| 10 | #8 OR #9 | 3,991,242 |
| 11 | #7 AND #10 | 3,165 |
| 12 | #11 AND [2003-2023]/py | 2,922 |
| 13 | 'systematic review':ti OR 'systematic literature review':ti OR 'scoping review':ti OR 'meta-anly*':ti OR 'integrative review':ti OR 'integrative literature review':ti OR 'narrative review':ti OR 'narrative literature review':ti | 282,688 |
| 14 | #12 NOT #13 | 2,821 |
| 15 | #14 AND ('article'/it OR 'article in press'/it OR 'preprint'/it) | 2,062 |

Cinahl

| **#** | **Query** | **Limiters/Expanders** | **Results** |
| --- | --- | --- | --- |
|  |  | Additional limiter: Academic Journals | 901 |
| 14 | S12 NOT 13 | Limiters - Published Date: 20030101-20231231  Expanders - Apply equivalent subjects  Search modes - Boolean/Phrase | 1,025 |
| 13 | TI "Systematic review" OR "Systematic literature review" OR "Scoping review" OR "Meta-anly*" OR "Integrative review" OR "Integrative literature review" OR "Narrative review" OR "Narrative literature review" | Limiters - Published Date: 20030101-20231231 Expanders - Apply equivalent subjects Search modes - Boolean/Phrase | 122,313 |
| S12 | S7 AND S10 | Limiters - Published Date: 20030101-20231231 Expanders - Apply equivalent subjects Search modes - Boolean/Phrase | 1,061 |
| S11 | S7 AND S10 | Expanders - Apply equivalent subjects Search modes - Boolean/Phrase | 1,139 |
| S10 | S8 OR S9 | Expanders - Apply equivalent subjects Search modes - Boolean/Phrase | 1,030,455 |
| S9 | TI ( Nurs* or Doctor* or Physician* or "Health Care Provider*" or "Health Professional*" or Clinician* or "Health Practitioner*" or "Healthcare Worker*" or "Health Care Worker*" or paramedic* or "emergency medical dispatcher" ) OR AB ( Nurs* or Doctor* or Physician* or "Health Care Provider*" or "Health Professional*" or Clinician* or "Health Practitioner*" or "Healthcare Worker*" or "Health Care Worker*" or paramedic* or "emergency medical dispatcher" ) | Expanders - Apply equivalent subjects Search modes - Boolean/Phrase | 998,686 |
| S8 | (MH "Health Personnel") or (MH "Health Occupations") | Expanders - Apply equivalent subjects Search modes - Boolean/Phrase | 53,562 |
| S7 | S5 AND S6 | Expanders - Apply equivalent subjects Search modes - Boolean/Phrase | 2,384 |
| S6 | TI ( (Improv* or Enhanc* or Build* or Increas* or Develop* or Promot*) N5 (Intervention* or Program* or Train* or Behaviour or Behavior OR Strateg*) ) OR AB ( (Improv* or Enhanc* or Build* or Increas* or Develop* or Promot*) N5 (Intervention* or Program* or Train* or Behaviour or Behavior OR Strateg*) ) | Expanders - Apply equivalent subjects Search modes - Boolean/Phrase | 331,340 |
| S5 | S3 OR S4 | Expanders - Apply equivalent subjects Search modes - Boolean/Phrase | 12,639 |
| S4 | TI ( (Work* or Occupation or Employ* or Job or Career) N5 (passion* or resilien* or adapta* or cope* or coping or hardiness or motivat*) ) OR AB ( (Work* or Occupation or Employ* or Job or Career) N5 (passion* or resilien* or adapta* or cope* or coping or hardiness or motivat*) ) | Expanders - Apply equivalent subjects Search modes - Boolean/Phrase | 10,917 |
| S3 | S1 AND S2 | Expanders - Apply equivalent subjects Search modes - Boolean/Phrase | 2,242 |
| S2 | (MH "Job Satisfaction+") OR (MH "Employment+") OR (MH "Employment Status") | Expanders - Apply equivalent subjects Search modes - Boolean/Phrase | 103,909 |
| S1 | (MH "Hardiness") OR (MH "Social Adjustment") | Expanders - Apply equivalent subjects Search modes - Boolean/Phrase | 21,081 |

Web of Science

| **#** | **Search** | **Results** |
| --- | --- | --- |
| 9 | #6 NOT #7 and Article (Document Types) | 1,001 |
| 8 | #6 NOT #7 | 1,067 |
| 7 | TI=("Systematic review" OR "Systematic literature review" OR "Scoping review" OR "Meta-anly*" OR "Integrative review" OR "Integrative literature review" OR "Narrative review" OR "Narrative literature review") | 284,541 |
| 6 | (#3 AND #4) AND (PY=(2003-2023)) | 1,129 |
| 5 | #3 AND #4 | 1,163 |
| 4 | #1 AND #2 | 6,327 |
| 3 | TS=(nurs* OR doctor* OR physician* OR "health care provider*" OR "health professional*" OR clinician* OR "health practitioner*" OR "healthcare worker*" OR "health care worker*" OR paramedic* OR "emergency medical dispatcher'") | 1,221,935 |
| 2 | TS=((Improv* or Enhanc* or Build* or Increas* or Develop* or Promot*) NEAR/5 (Intervention* or Program* or Train* or Behaviour or Behavior OR Strateg*) ) | 1,447,851 |
| 1 | TS=((Work* or Occupation or Employ* or Job or Career) NEAR/5 (passion* or resilien* or adapta* or cope* or coping or hardiness or motivat*)) | 62,746 |
