# Supplementary material for: Interventions to promote resilience and passion for work in health settings: A mixed-methods systematic review
Source: Int J Nurs Stud Adv. 2024 Sep 21;7:100242. doi: 10.1016/j.ijnsa.2024.100242 (PMC11460621; doi:10.1016/j.ijnsa.2024.100242)
Supplement: Supplementary file 2 [file mmc2.docx]

**Supplementary online material (Appendix 2)**

**Summary of the quality assessment of the included studies using the Mixed Methods Appraisal Tool (MMAT) version 2018 (n = 33)**

1. **Qualitative (n = 4)**

| **Methodological quality criteria** | **Foster et al., 2018** | **McDonald et al., 2012** | **McDonald et al., 2013** | **Potter et al., 2015** |
| --- | --- | --- | --- | --- |
| S1. Are there clear research questions? | √ | √ | √ | √ |
| S2. Do the collected data allow to address the research questions? | √ | √ | √ | √ |
| 1.1. Is the qualitative approach appropriate to answer the research question? | √ | √ | √ | √ |
| 1.2. Are the qualitative data collection methods adequate to address the research question? | √ | √ | √ | √ |
| 1.3. Are the findings adequately derived from the data? | √ | √ | √ | √ |
| 1.4. Is the interpretation of results sufficiently substantiated by data? | √ | √ | √ | √ |
| 1.5. Is there coherence between qualitative data sources, collection, analysis and interpretation? | √ | √ | √ | √ |

*Note*: “S” = Screening question, “√” = yes; “X” = no; “?” = cannot tell

1. **Quantitative randomized controlled trials (n=11)**

| **Methodological quality criteria** | **Chesak et al., 2015** | **Duva et al., 2022** | **Dyrbye et al., 2019** | **Grabbe et al., 2020** | **Henshall et al., 2023** | **Lin et al., 2019** | **Mache et al., 2015** | **Mao et al., 2021** | **Mealer et al., 2014** | **Slatyer et al., 2018** | **Sood et al., 2011** |
| --- | --- | --- | --- | --- | --- | --- | --- | --- | --- | --- | --- |
| S1. Are there clear research questions? | √ | √ | √ | √ | √ | √ | √ | √ | √ | √ | √ |
| S2. Do the collected data allow to address the research questions? | √ | √ | √ | √ | √ | √ | √ | √ | √ | √ | √ |
| 2.1. Is randomization appropriately performed? | √ | √ | √ | √ | √ | √ | √ | √ | √ | √ | √ |
| 2.2. Are the groups comparable at baseline? | √ | √ | √ | √ | √ | √ | √ | √ | √ | √ | √ |
| 2.3. Are there complete outcome data? | X | √ | √ | √ | √ | √ | √ | √ | X | X | √ |
| 2.4. Are outcome assessors blinded to the intervention provided? | ? | ? | ? | √ | √ | √ | ? | ? | √ | √ | ? |
| 2.5 Did the participants adhere to the assigned intervention? | √ | X | √ | X | √ | √ | ? | √ | √ | X | √ |

*Note*: “S” = Screening question, “√” = yes; “X” = no; “?” = cannot tell.

1. **Quantitative non-randomized (n = 14)**

| **Methodological quality criteria** | **Babanataj et al., 2018** | **Blackburn et al., 2020** | **Bonamer & Aquino-Russell, 2019** | **Craigie et al., 2016** | **Detore et al., 2022** | **Fortney et al., 2013** | **Franco & Christie, 2021** |
| --- | --- | --- | --- | --- | --- | --- | --- |
| S1. Are there clear research questions? | √ | √ | √ | √ | √ | √ | √ |
| S2. Do the collected data allow to address the research questions? | √ | √ | √ | √ | √ | √ | √ |
| 3.1. Are the participants representative of the target population? | √ | ? | √ | √ | √ | √ | √ |
| 3.2. Are measurements appropriate regarding both the outcome and intervention (or exposure)? | √ | √ | √ | √ | √ | √ | √ |
| 3.3. Are there complete outcome data? | √ | √ | X | X | X | X | X |
| 3.4. Are the confounders accounted for in the design and analysis? | ? | √ | ? | √ | ? | ? | ? |
| 3.5. During the study period, is the intervention administered (or exposure occurred) as intended? | √ | √ | √ | √ | √ | √ | √ |

*Note*: “S” = Screening question, “√” = yes; “X” = no; “?” = cannot tell

1. **Quantitative non-randomized (n = 14)** **(Continued)**

| **Methodological quality criteria** | **Haugland et al., 2023** | **Kelly et al., 2021** | **Magtibay et al., 2017** | **Mintz-Binder et al., 2021** | **Muir et al., 2022** | **Rushton et al., 2021** | **Yi-Frazier et al., 2022** |
| --- | --- | --- | --- | --- | --- | --- | --- |
| S1. Are there clear research questions? | √ | √ | √ | √ | √ | √ | √ |
| S2. Do the collected data allow to address the research questions? | √ | √ | √ | √ | √ | √ | √ |
| 3.1. Are the participants representative of the target population? | √ | √ | √ | ? | √ | ? | √ |
| 3.2. Are measurements appropriate regarding both the outcome and intervention (or exposure)? | √ | √ | √ | √ | √ | √ | √ |
| 3.3. Are there complete outcome data? | X | X | √ | ? | X | X | √ |
| 3.4. Are the confounders accounted for in the design and analysis? | ? | X | X | ? | ? | ? | X |
| 3.5. During the study period, is the intervention administered (or exposure occurred) as intended? | √ | √ | X | √ | ? | ? | X |

*Note*: “S” = Screening question, “√” = yes; “X” = no; “?” = cannot tell

1. **Mixed methods (n = 4)**

| **Methodological quality criteria** | **Crandall et al., 2022** | **Delaney, 2018** | **Henshall et al., 2020** | **Kim et al., 2022** |
| --- | --- | --- | --- | --- |
| S1. Are there clear research questions? | √ | √ | √ | √ |
| S2. Do the collected data allow to address the research questions? | √ | √ | √ | √ |
| 5.1. Is there an adequate rationale for using a mixed methods design to address the research question? | √ | √ | √ | ? |
| 5.2. Are the different components of the study effectively integrated to answer the research question? | √ | √ | √ | √ |
| 5.3. Are the outputs of the integration of qualitative and quantitative components adequately interpreted? | ? | √ | √ | √ |
| 5.4. Are divergences and inconsistencies between quantitative and qualitative results adequately addressed? | √ | √ | √ | √ |
| 5.5. Do the different components of the study adhere to the quality criteria of each tradition of the methods involved? | √ | √ | √ | √ |

*Note*: “S” = Screening question, “√” = yes; “X” = no; “?” = cannot tell.
