# Supplementary material for: Interventions to promote resilience and passion for work in health settings: A mixed-methods systematic review
Source: Int J Nurs Stud Adv. 2024 Sep 21;7:100242. doi: 10.1016/j.ijnsa.2024.100242 (PMC11460621; doi:10.1016/j.ijnsa.2024.100242)
Supplement: Supplementary file 4 [file mmc4.pdf]

Interventions to promote resilience and passion in health settings: A mixed-methods systematic review

To enable PROSPERO to focus on COVID-19 submissions, this registration record has undergone basic automated checks for eligibility and is published exactly as submitted. PROSPERO has never provided peer review, and usual checking by the PROSPERO team does not endorse content. Therefore, automatically published records should be treated as any other PROSPERO registration. Further detail is provided here.

Citation

supan unjai, Elizabeth Forster, Amy Mitchell, Debra K Creedy. Interventions to promote resilience and passion in health settings: A mixed-methods systematic review. PROSPERO 2023 CRD42023394575 Available from:
https://www.crd.york.ac.uk/prospero/display_record.php?ID=CRD42023394575

Review question

Research questions will guide the review:

1. What are the characteristics of interventions used to promote resilience or passion for work in health settings?

2. What is the effectiveness of interventions used to promote resilience or passion for work in health settings?

Searches

Six electronic bibliographic databases will be used including MEDLINE (via Ovid), EMBASE, CINAHL (via EBSCOhost), Web of Science, Scopus, and PsycINFO (via Ovid). We will hand search the reference lists of eligible studies for any other potentially eligible studies that were not already captured by the database searches.

The review team developed search terms with a librarian as following.

1) Coping Behavior [MeSH] or "Resilience (Psychological)" [MeSH] or Social Adjustment [MeSH]

2) Quality of Work Life"[MeSH] or Employment Status [MeSH]

3) (Work* or Occupation or Employ* or Job or Career) adj5 (passion* or resilien* or adapta* or cope* or coping or hardiness or motivat*)

4) (Improv* or Enhanc* or Build* or Increas* or Develop* or Promot*) adj5 (Intervention* or Program* or Train* or Behaviour or Behavior or Strateg*)

5) nurs* or doctor* or physician* or “health care provider*” or "health professional*" or clinician* or “health practitioner*” or healthcare or hospital or “health service” or “healthcare worker*” or “health facilities” or medicine or medical or health

The search will be limited to papers published between 2003 and current, written in English, and published in peer reviewed journals.

The Preferred Reporting Items for Systematic reviews and Meta-Analyses (PRISMA) revised version 2020 will be used for reporting of reviews evaluating the effectiveness of interventions (Page et al., 2021).

Types of study to be included

Any type of study will be included: quantitative, qualitative, and mixed-methods studies inclusive of experimental study designs, including randomised controlled trials, non-randomised controlled trials, and quasi-experimental or observational study designs (e.g., cohort studies, cross-sectional studies, and case-control studies) that evaluate the effectiveness of interventions designed to promote resilience and passion for work in health professional occupational groups. Included studies must be published in English and peer-reviewed journals.

Condition or domain being studied

Any interventions to enhance resilience or passion for work in health settings.

Participants/population

Inclusion: All workers in health occupations

Exclusion: Studies focused on adult workers in non-professional occupations.

Studies where interventions are specifically designed for or delivered to workers who are identified as being in the clinical range on a measure or having a diagnosed mental health condition.

Intervention(s), exposure(s)

A key aspect of this review is to identify interventions used to promote resilience and/or passion for health professional work. These interventions may be diverse in terms of underpinning theoretical approach and be designed for individuals or groups, they may be delivered via diverse modalities including face-to-face, online or using an integrated approach. Studies will be included if they target workers in health settings. The studies that do not explicitly relate resilience or passion in 'work' or 'job' or 'occupation' (e.g., exercising, playing games, being with illness, etc.) will be excluded.

Comparator(s)/control

Any comparison group (e.g. wait-list, education, non-exposed control) or no comparison group (e.g. pre-post study designs)

Context

Studies focusing on the intervention used to promote resilience and/or passion relevant to work and conducted in any health setting or workplace will be included.

Main outcome(s)

The primary outcomes are 1) the characteristics or details of interventions used to promote resilience or passion for work and 2) effectiveness of the interventions used to promote resilience or passion for work, such a change in resilience and passion for work scores, the experiences of participants after receiving an intervention (e.g., perception, experiences, and attitudes).

Additional outcome(s)

None

Data extraction (selection and coding)

Literature search results from databases and other sources will be screened by title and abstract, using Rayyan software, by two reviewers. Two reviewers will screen the full texts of potentially eligible studies. A discussion with a third

reviewer will decide any differences of opinion. Missing data will be requested from the study authors.

The review team members will develop a data extraction template, which includes author, year, country, objective, study design, participant characteristics, sample size, setting, intervention details, comparator and duration, intervention evaluation, outcomes, and critique.

Risk of bias (quality) assessment

The Mixed Methods Appraisal Tool version 2018 (Hong et al, 2018) will be used to assess the quality of five study designs: qualitative, randomized controlled, nonrandomized, quantitative descriptive, and mixed methods.

Strategy for data synthesis

This review will follow a convergent synthesis design for data synthesis (Hong et al., 2017). In this design, quantitative and qualitative data are analyzed separately using the same or different synthesis methods and then both results are combined using narrative synthesis.

For qualitative data, narrative synthesis will be used to summarize and explain the findings of included studies.

For quantitative data, results from studies using comparable outcome measures will be combined using meta-analysis to generate a quantitative estimate of the efficacy/effectiveness of interventions. If there are insufficient RCTs to conduct meta-analysis, quantitative data will be synthesized narratively.

Analysis of subgroups or subsets

Where possible there will be a separate presentation, exploration and/or analysis of the characteristics of interventions used with different groups of participants (e.g., health care providers); different types of interventions (e.g., personal coaching, psychoeducation, clinical supervision); different settings (e.g., country, inpatient, outpatient, community; and different types of study (e.g., randomised or non-randomised trials).

Contact details for further information

supan unjai

supan.unjai@griffithuni.edu.au

Organisational affiliation of the review

Griffith University

https://www.griffith.edu.au/

Review team members and their organisational affiliations

Mrs supan unjai. PhD candidate at School of Nursing and Midwifery, Nathan Campus, Griffith University, Australia.

Assistant/Associate Professor Elizabeth Forster. Program Director Postgraduate Nursing & Infection Prevention and Control Program Advisor Paediatric Nursing, School of Nursing and Midwifery, Nathan Campus, Griffith University, Australia.

Dr Amy Mitchell. Lecturer, School of Nursing and Midwifery, Nathan Campus, Griffith University, Australia.

Professor Debra K Creedy. School of Nursing & Midwifery, Logan Campus, Australia

Type and method of review

Systematic review, Other

Anticipated or actual start date

01 February 2023

Anticipated completion date

30 June 2023

Funding sources/sponsors

none

Conflicts of interest

Language

English

Country

Australia

Stage of review

Review Ongoing

Subject index terms status

Subject indexing assigned by CRD

Subject index terms

Emotions; Humans; Research Design; Resilience, Psychological

Date of registration in PROSPERO

05 February 2023

Date of first submission

26 January 2023

Details of any existing review of the same topic by the same authors

None

Stage of review at time of this submission

The review has not started

Preliminary searches	No	No
Piloting of the study selection process	No	No
Formal screening of search results against eligibility criteria	No	No
Data extraction	No	No
Risk of bias (quality) assessment	No	No
Data analysis	No	No

The record owner confirms that the information they have supplied for this submission is accurate and complete and they understand that deliberate provision of inaccurate information or omission of data may be construed as scientific misconduct.

The record owner confirms that they will update the status of the review when it is completed and will add publication details in due course.

Versions

05 February 2023

05 February 2023
